# The Determinants of Against Medical Advice Hospital Discharges

**DOI:** 10.7759/cureus.97691

**Published:** 2025-11-24

**Authors:** Aditya Kaliath, Jamie L Romeiser, Aaron Shaykevich, Ivayla I Geneva

**Affiliations:** 1 Department of Public Health and Preventive Medicine, State University of New York Upstate Medical University, Syracuse, USA; 2 Department of Internal Medicine, Crouse Hospital, Syracuse, USA

**Keywords:** alcohol dependence, discharge against medical advice (ama), mental health, social deprivation index, social determinants of health (sdoh), substance dependence

## Abstract

Background: Discharges against medical advice (AMA) are associated with adverse patient outcomes, higher readmission rates, and increased hospital expenses. Understanding the determinant of AMA is expected to guide the development of preventive measures.

Methods: A retrospective cohort study comparing adult inpatient AMA and non-AMA discharges at SUNY Upstate Medical University Hospital over a one-year period. Data was analyzed at-the-person as well as at-the-hospitalization level. The independent variables studied were age, race, sex, mental health, substance use, alcohol use, Social Deprivation Index (SDI), length of stay (LOS), season, and history of prior AMA. Fisher’s exact test, Chi-square tests, and Wilcoxon rank sum tests were used for descriptive and bivariate analyses, logistic regression, and generalized estimating equation (GEE) models for multivariate analyses.

Results: At-the-person level, increased risk for AMA (p < 0.05) was associated with being male, African-American, young adult, having a history of substance or alcohol use, and higher SDI. At-the-hospitalization level, AMA discharges featured shorter LOS, prior AMA, substance use, or alcohol use. Adjusted logistic regression identified male sex, African-American race, substance use, alcohol use, younger age, and SDI ≥ 80 as independent predictors of AMA discharge at-the-person level. GEE models yielded similar results at-the-hospitalization level, with prior AMA history being the strongest risk factor.

Conclusion: AMA discharges present a significant challenge for hospitals and compromise patient care. This study identified several key risk factors predictive of AMA. Knowledge of the determinants of AMA would allow for early identification of at-risk patients and for potential early interventions to prevent AMA discharges.

## Introduction

Patients leaving the hospital before medical treatment is complete, known as leaving against medical advice (AMA), has been an ongoing challenge in healthcare. For decades, 1-2% of all hospital discharges in the United States are AMA [1, 2], and these are associated with higher patient morbidity and mortality as well as higher rates of 30-day hospital readmissions [3]. As a result, AMA discharges result in a significant financial burden to the healthcare system [4,5]. Despite their clinical and financial consequences, the reasons why patients leave AMA remain complex and multifactorial. Some studies have investigated the role of mental health conditions such as depression, anxiety, and substance use disorders [2,6,7], while others have emphasized the importance of socioeconomic pressures, dissatisfaction with care, logistical issues such as wait times, and even systemic biases [8,9]. Environmental and temporal factors have also been suggested to influence AMA behavior. Qualitative studies have reported weather-related stressors, transportation challenges, and seasonal obligations (e.g., housing instability or work conflicts) as contributing factors [10,11]. Though rarely quantified, such influences may help explain observed temporal trends, such as the rising rates of AMA discharges over the past two decades in patient subgroups [7,12]. Yet, seasonality itself remains an underexplored risk factor. Despite this growing awareness, few studies have taken a comprehensive approach to evaluating multiple domains of risk in a single analysis. Current literature tends to focus on narrow patient subgroups or isolated risk factors. For example, AMA in patients with gastrointestinal bleeding [13], sickle cell disease [9], inflammatory bowel disease [12], dermatologic conditions [14], or pancreatitis [7], minimizing the generalizability of findings. Furthermore, there is limited research that distinguishes between patient-level risk (i.e., which types of patients are likely to ever go AMA) and hospitalization-level risk (i.e., under what conditions AMA events occur). Our study aimed to evaluate a broad range of demographic, clinical, psychosocial, and logistical factors associated with AMA discharges among hospitalized patients. By better understanding the multifactorial causes of AMA discharges, this research aims to provide a foundation for targeted prevention strategies and improved continuity of care.

## Materials and methods

This retrospective cohort study aimed to identify the determinants of AMA discharges at the State University of New York Upstate Medical University Hospital in Syracuse, NY, USA, evaluating hospitalizations between June 1, 2023, and May 31, 2024. Data were collected from the EPIC electronic health record system (Epic Systems Corporation, 2024) using EPIC’s data extraction tool SlicerDicer. The study population included all adult patients (age ≥ 18 years) admitted to the hospital during this time frame. Each hospitalization was noted to result in either an “AMA” discharge or in a “non-AMA” discharge, which constituted our dependent variable. To examine for a potential seasonal variation in AMA discharges, we noted the season during which the patient was discharged (summer: June 1, 2023-August 31, 2023; fall: September 1, 2023-November 30, 2023; winter: December 1, 2023-February 29, 2024; and spring: March 1, 2024-May 31, 2024. We included patients’ demographics (age, sex, race). We used the zip codes associated with patients’ residence to derive each patient’s Social Deprivation Index (SDI) using the ZIP Code Tabulation Areas in the 2017-2022 dataset from the Robert Graham Center [15,16], accessed on September 11, 2024. The SDI is a composite measure of area-level disadvantage based on income, education, employment, housing, and other social determinants, with scores ranging from 0 to 100, with higher scores indicating greater social deprivation. We categorized the SDI into quintiles (0-19, 20-39, 40-59, 60-79, and 80+). We noted the presence of mental health history (ICD-10 psychiatric diagnoses (anxiety disorders, mood disorders, personality disorders, eating disorders, post-traumatic stress disorder, psychotic disorders), intellectual disabilities, and dementia-related conditions), substance abuse (all ICD-10 substance use diagnoses except alcohol abuse), and alcohol dependence [17]. We also noted the length of stay (LOS) for each hospitalization (divided into categories: <1 day, 1-2 days, 2-8 days, 8-25 days, and more than 25 days). We noted whether each patient had a history of AMA discharge during the previous 12 months.

All statistical analyses were performed using SAS software, Version 9.4 (Released: 2013; SAS Institute Inc., Cary, NC, USA), with statistical significance assessed at the 0.05 level. Missing data were minimal (less than 1% of the total) except for the zip code variable that was used to create the SDI variable as described above, where the missing data amounted to 14% (total n = 21,490 and missing data for n = 3,009). Models were run with a “missing” category and with listwise deletion. There was minimal difference between the effects for both models, and no difference in conclusions; therefore, final models were run with the listwise deletion for missing data.

Data were analyzed in two ways: at-the-person level and at-the-hospitalization level. Starting with the at-the-person level approach, we divided the patients into two groups: the patients who had never left AMA during the study period and the patients who had left AMA at least once during the study period. This definition meant that a portion of the hospitalizations were excluded; namely, if the same patient had an AMA discharge and a non-AMA discharge during the study period, only the chronologically first hospitalization associated with an AMA discharge was included in the analyses. Here, we used only the independent variables that could be assumed to have remained constant throughout the study period (race, age, sex, mental health diagnosis, substance use, alcohol use, and SDI). For the at-the-hospitalization level approach, we included all hospitalizations of all patients during the study period. Here, each hospitalization served as a unique unit of analysis and was classified as AMA or non-AMA based on its discharge disposition. In addition to the abovementioned independent variables, here, we also included the season during which the discharge occurred, LOS, and “history of AMA discharge” over the previous 12 months.

Person-level bivariate analyses were performed using Fisher's exact tests or Chi-square tests for categorical variables. Continuous data were examined for normality (Shapiro-Wilk < 0.05) and analyzed using Wilcoxon rank sum tests. Data were further analyzed with unadjusted and adjusted logistic regression models. Visit-level data were analyzed using generalized estimating equation (GEE) models with a logit link to account for repeated measures. For multivariable models, SDI and median income were found to be highly correlated; therefore, median income was dropped from the model. Odds ratios (OR) are reported for both unadjusted and adjusted models. All analyses were performed at the 0.05 significance level using SAS software.

## Results

Descriptive statistics and bivariate analysis for person-level variables

Regarding the data at-the-person level (Table 1), we included a total of 21,490 patients admitted to SUNY Upstate Medical University Hospital between June 1, 2023, and May 31, 2024. Overall, 717 (3.3%) of the patients left the hospital AMA at least once, while 20,773 (96.7%) did not during the study period. Males were found to have a higher AMA rate of 4.2% (435 out of 10,414) as opposed to females having a rate of 2.5% (282 out of 11,075), with p < 0.0001. Regarding patients’ race, Black or African American patients featured the highest AMA rate of 6.9% (184 out of 2,656), followed by 4.8% (11 out of 230) for American Indian or Alaska Native, with p < 0.0001. Patients with mental health diagnoses had a higher rate of AMA at 4.3% (97 out of 2,260) vs 3.2% (620 out of 19,230) for patients without such a diagnosis, with p < 0.001. Patients with a history of substance abuse were also more likely to leave AMA at a rate of 22.8% (53 out of 232) versus those without such a diagnosis at a rate of 3.1% (664 out of 21,258), with p < 0.0001. Alcohol abuse was also associated with a higher AMA rate of 16.1% (31 out of 193) compared to patients without this diagnosis, who featured an AMA rate of 3.2% (686 out of 21,297), with p < 0.0001. The median age of all patients was 62 years (interquartile range (IQR): 42-75). Among those who left AMA, the median age was notably smaller at 48 years (IQR: 36-62), compared to 63 years (IQR: 43-75) for patients who did not leave AMA. Rates of AMA increased with age from the 18-24 age range at 2.8% (39 out of 1,386) until the 35-44 age range at 6.5% (151 out of 2,339), and then decreased as age increased with a rate of 1.5% (148 out of 9,689) in the >65 years old category, which were found to be statistically significant with p < 0.001. A patient with a high SDI was more than twice as likely to leave AMA at a rate of 5.7% (339 out of 5,896) compared to a patient with a lower SDI, who featured a rate of 2.5% (303 out of 12,193), with p < 0.0001. When further subdivided by quintiles, the rate of patients leaving AMA increases significantly from 1.8% (75 out of 4,093) at the lowest SDI up to 6.4% (292 out of 4,547) with an SDI of > 80. This is congruent with the finding of the median income of patients having left AMA being $59495.0 as opposed to the median income of patients not having ever left AMA being $67672.0 (p < 0.0001).

**Table 1:** Person-level characteristics of patients by AMA discharge status-descriptive statistics and bivariate analysis AMA: against medical advice; SDI: Social Deprivation Index; WRS: Wilcoxon rank sum test; Chisq: Chi-square test; Fisher: Fisher’s exact test

Variable	All	Any AMA during the study period	No AMA during the study period	p-value	Test (statistic)
Total patient count	21490	717 (3.3%)	20773 (96.7%)		
Race				<0.0001	Fisher (129.9)
American Indian or Alaska Native	230 (1.1%)	11 (4.8%)	219 (95.2%)		
Asian/Asian Indian	173 (0.8%)	2 (1.2%)	171 (98.8%)		
Black or African American	2656 (12.4%)	184 (6.9%)	2472 (93.1%)		
Other	990 (4.6%)	39 (3.9%)	951 (96.1%)		
Unknown	213 (1%)	4 (1.9%)	209 (98.1%)		
White or Caucasian	17228 (80.2%)	477 (2.8%)	16751 (97.2%)		
Sex				<0.0001	Chisq (44.3)
Female	11075 (51.5%)	282 (2.5%)	10793 (97.5%)		
Male	10414 (48.5%)	435 (4.2%)	9979 (95.8%)		
Mental health				0.001	Chisq (7.2)
No	19230 (89.5%)	620 (3.2%)	18610 (96.8%)		
Yes	2260 (10.5%)	97 (4.3%)	2163 (95.7%)		
None of the above	7631 (35.5%)	261 (3.4%)	7370 (96.6%)		
Alcohol use				<0.0001	
No alcohol use	21297 (99.1%)	686 (3.2%)	20611 (96.8%)		Chisq (97.8)
Alcohol use	193 (0.9%)	31 (16.1%)	162 (83.9%)		
Substance use				<0.0001	
No substance use	21258 (98.9%)	664 (3.1%)	20594 (96.9%)		Chisq (276.6)
Substance use present	232 (1.1%)	53 (22.8%)	179 (77.2%)		
Any comorbidity (mental health, alcohol use, substance use)				<0.0001	Chisq (73.5)
No comorbidities	18942 (88.1%)	559 (3%)	18383 (97%)		
Has ≥1 comorbidity	2548 (11.9%)	158 (6.2%)	2390 (93.8%)		
First age (median, Q1, Q3)	62 (42, 75)	48 (36, 62)	63 (43, 75)	0.6	WRS (175.3)
18-24	1386 (6.4%)	39 (2.8%)	1347 (97.2%)	<0.0001	Chisq (222.9)
25-34	2200 (10.2%)	122 (5.5%)	2078 (94.5%)		
35-44	2339 (10.9%)	151 (6.5%)	2188 (93.5%)		
45-64	5876 (27.3%)	257 (4.4%)	5619 (95.6%)		
65+	9689 (45.1%)	148 (1.5%)	9541 (98.5%)		
High SDI					
No	12193 (67.4%)	303 (2.5%)	11890 (97.5%)	<0.0001	Chisq (123.7)
Yes	5896 (32.6%)	339 (5.7%)	5557 (94.3%)		
SDI				<0.0001	Chisq (158.6)
0-19	4093 (22.6%)	75 (1.8%)	4018 (98.2%)		
20-39	2219 (12.3%)	65 (2.9%)	2154 (97.1%)		
40-59	3107 (17.2%)	78 (2.5%)	3029 (97.5%)		
60-79	4123 (22.8%)	132 (3.2%)	3991 (96.8%)		
80+	4547 (25.1%)	292 (6.4%)	4255 (93.6%)		
Median income (median, Q1, Q3)	67614 (57503, 90315)	59495 (40641, 74931)	67672 (58946, 90315)	<0.0001	WRS (140.1)

Descriptive statistics and bivariate analysis for hospitalization-level variables

Regarding the data at-the-hospitalization level (Table 2), we included a total of 30,937 hospital visits during the same study period, of which 2.7% left AMA. Shorter LOS had higher AMA rates. AMA events were highest for visits 1-2 days long at 6.5%, followed by visits less than one day at 5.3%. Any LOS longer than two days accounted for 4.5% of AMA ending visits (p < 0.0001). There did not appear to be a correlation between seasonality and visits ending in AMA (p > 0.05). Finally, a patient having a prior history of having left AMA was strongly associated with AMA ending visits (p < 0.0001).

**Table 2:** Hospitalization-level characteristics associated with AMA discharge status-descriptive statistics and bivariate analysis AMA: against medical advice; SDI: Social Deprivation Index; GEE: generalized estimating equation

Variable	All		AMA	No AMA	p-value	Test (statistic)
Total patient count	30937		846 (2.7%)	30091 (97.3%)		
Length of stay					<0.0001	GEE (154.6)
2.0-7.9 days	3348 (10.8%)		179 (5.3%)	3169 (94.7%)		
1-2.0 days	3919 (12.7%)		256 (6.5%)	3663 (93.5%)		
2.0-7.9 days	16476 (53.3%)		308 (1.9%)	16168 (98.1%)		
8.0-24.9 days	6300 (20.4%)		93 (1.5%)	6207 (98.5%)		
≥25.0 days	894 (2.9%)		10 (1.1%)	884 (98.9%)		
Season					0.90	GEE (0.57)
Fall	7779 (25.1%)		211 (2.7%)	7568 (97.3%)		
Spring	7798 (25.2%)		206 (2.6%)	7592 (97.4%)		
Summer	7791 (25.2%)		218 (2.8%)	7573 (97.2%)		
Winter	7569 (24.5%)		211 (2.8%)	7358 (97.2%)		
Prior AMA during time examined					<0.0001	GEE (86.4)

Logistic regression analysis for person-level variables

On the personal level analysis, race, gender, substance use, alcohol use, age, and SDI were all significant predictors of patients leaving AMA (Table 3). It should be noted that although in the tables we report both unadjusted and adjusted OR, hereafter, we will only refer to the adjusted ORs and abbreviate them as “OR”. Black or African American patients had the highest likelihood of leaving AMA with an OR of 1.52 (95% CI: 1.23-1.87; p = 0.0001). Males were 1.76 times more likely to leave AMA than females (95% CI: 1.49-2.08; p < 0.0001). A history of substance or alcohol abuse was a strong predictor of AMA being 5.83 (95% CI: 4.13-8.22; p = 0.0001) and 4.50 (95% CI: 2.94-6.90; p = 0.0001) times more likely, respectively, to leave AMA. The likelihood of leaving AMA increased with age brackets up to the 35-44 age group, having the highest OR of 3.35 (95% CI: 2.61-4.32; p < 0.0001), and then declining with a drop off in the 65+ group (OR = 1). Finally, SDI was directly correlated with AMA, as the higher the SDI, the higher the likelihood of leaving AMA. Patients with an SDI of 80 or greater were 2.35 (95% CI: 1.78-3.11; p < 0.0001) times more likely to leave AMA compared to those with an SDI of 0-19. Median income was not included in the analysis as it is accounted for in the SDI.

**Table 3:** Unadjusted and adjusted logistic regression analysis for person-level predictors of AMA discharge AMA: against medical advice; SDI: Social Deprivation Index; OR: odds ratio; LCL: lower confidence limit; UCL: upper confidence limit; ref: reference

Variable	Unadjusted models					Adjusted models			
	Unadjusted OR	LCL	UCL	p-value		Adjusted OR	LCL	UCL	p-value
Race									
American Indian or Alaska Native	1.76	0.96	3.25	0.07		1.34	0.71	2.56	0.37
Asian/Asian Indian	0.41	0.10	1.66	0.21		0.30	0.07	1.22	0.09
Black or African American	2.61	2.19	3.12	< .0001>		1.52	1.23	1.87	0.0001
Other	1.44	1.03	2.01	0.03		0.84	0.57	1.22	0.35
Unknown	0.67	0.25	1.82	0.43		0.55	0.17	1.75	0.31
White or Caucasian	ref	ref	ref	ref					
Sex (male vs. female)	1.67	1.43	1.94	< .0001>		1.76	1.49	2.08	< .0001>
Mental health (yes vs. no)	1.35	1.08	1.68	0.01		1.12	0.88	1.42	0.34
Substance use (yes vs. no)	9.18	6.70	12.60	< .0001>		5.83	4.13	8.22	< .0001>
Alcohol use (yes vs. no)	5.75	3.89	8.51	< .0001>		4.50	2.94	6.90	< .0001>
Age category									
18-24	1.87	1.31	2.67	0.04		1.53	1.03	2.28	0.04
25-34	3.79	2.96	4.83	< .0001>		2.88	2.20	3.76	< .0001>
35-44	4.45	3.53	5.61	< .0001>		3.35	2.61	4.32	< .0001>
45-64	2.95	2.40	3.62	0.01		2.27	1.82	2.83	< .0001>
65+	ref	ref	ref	ref		ref	ref	ref	ref
SDI									
0-19	ref	ref	ref	ref		ref	ref	ref	ref
20-39	1.62	1.16	2.26	0.01		1.37	0.97	1.93	0.07
40-59	1.38	1.00	1.90	0.05		1.21	0.87	1.67	0.26
60-79	1.77	1.33	2.36	< .0001>		1.45	1.09	1.95	0.01
80+	3.68	2.84	4.76	< .0001>		2.35	1.78	3.11	< .0001>
Median income (per 10,000)	0.80	0.77	0.83	< .0001>		-	-	-	-

Logistic regression analysis for hospitalization-level variables

Similar to personal-level findings, a history of prior AMA, LOS, age, SDI, and substance use were all predictors of AMA at the hospitalization level (Table 4). The Black or African American race had the strongest association with AMA, with a 1.44 likelihood of leaving AMA (95% CI: 1.14-1.80; p = 0.002) compared to White patients. At the hospitalization level, males were 1.74 times more likely (95% CI: 1.46-2.07; p < 0.0001) than females to leave AMA. As with personal-level findings, substance abuse had a strong predictive value (OR = 3.80; 95% CI: 2.47-5.86; p < 0.0001) as well as alcohol abuse (OR = 2.42; 95% CI: 1.38-4.24; p = 0.001) with AMA. Regarding LOS, 1-2 days appeared to have the highest correlation with AMA. Less than 1 or more than 2 days were less likely to be associated with AMA. As with the personal level, the 35-44 age category was the most likely to leave AMA (OR = 3.12; 95% CI: 2.36-4.13; p < 0.0001) of the age groups, with those older than 65 being the least. Again, same as with the personal level, a history of having left AMA was strongly associated with leaving AMA again. Finally, SDI followed a similar pattern of increasing alongside the likelihood of leaving AMA, with greater than 80 years old having an OR of 1.98 (95% CI: 1.49-2.63; p < 0.0001).

**Table 4:** Unadjusted and adjusted GEE logistic regression analysis for hospitalization-level predictors of AMA discharge AMA: against medical advice; SDI: Social Deprivation Index; GEE: generalized estimating equation; OR: odds ratio; LCL: lower confidence limit; UCL: upper confidence limit; ref: reference

Variable	Unadjusted models					Adjusted models			
	Unadjusted OR	LCL	UCL	p-value		Adjusted OR	LCL	UCL	p-value
Rac									
American Indian or Alaska Native	1.67	0.89	3.12	0.11		1.24	0.64	2.41	0.53
Asian/Asian Indian	0.40	0.10	1.61	0.20		0.34	0.08	1.41	0.14
Black or African American	2.34	1.95	2.79	< .0001>		1.44	1.14	1.80	0.002
Other	1.19	0.85	1.67	0.32		0.94	0.64	1.37	0.73
Unknown	0.27	0.10	0.72	0.01		0.89	0.29	2.75	0.84
White or Caucasian	ref	ref	ref	ref		ref	ref	ref	ref
Sex (male vs. female)	1.62	1.39	1.90	< .0001>		1.74	1.46	2.07	< .0001>
Mental Health (yes vs. no)	1.06	0.84	1.35	0.60		0.78	0.60	1.02	0.07
Substance (yes vs. no)	10.51	7.70	14.34	< .0001>		3.80	2.47	5.86	< .0001>
Alcohol (yes vs. no)	5.64	3.78	8.41	< .0001>		2.42	1.38	4.24	0.002
Length of stay									
<1 day	0.68	0.54	0.86	0.00		0.77	0.61	0.96	0.022
1-2 days	ref	ref	ref	ref		ref	ref	ref	ref
2.0 days or more and less than 8.0 days	0.32	0.26	0.40	< .0001>		0.27	0.23	0.33	< .0001>
8.0 days or more and less than 25.0 days	0.29	0.21	0.38	< .0001>		0.23	0.17	0.30	< .0001>
25.0 days or more	0.19	0.08	0.44	0.0001		0.14	0.06	0.31	< .0001>
Age category									
18-24	2.26	1.57	3.26	< .0001>		1.63	1.10	2.42	0.02
25-34	4.43	3.45	5.70	< .0001>		2.75	2.05	3.68	< .0001>
35-44	4.91	3.87	6.23	< .0001>		3.12	2.36	4.13	< .0001>
45-64	3.18	2.57	3.92	< .0001>		2.23	1.76	2.83	< .0001>
65+	ref	ref	ref	ref		ref	ref	ref	ref
Not first AMA (yes vs. no)	28.17	21.99	36.09	< .0001>		12.01	9.17	15.72	< .0001>
SDI									
0-19	ref	ref	ref	ref		ref	ref	ref	ref
20-39	1.56	1.10	2.23	0.01		1.32	0.94	1.85	0.10
40-59	1.42	1.01	1.99	0.04		1.13	0.80	1.59	0.50
60-79	1.86	1.37	2.52	< .0001>		1.51	1.13	2.01	0.005
80+	3.55	2.71	4.67	< .0001>		1.98	1.49	2.63	< .0001>
Median income (per 10,000)	0.80	0.77	0.84	< .0001>		-	-	-	-

## Discussion

This study analyzed more than 30,000 hospitalizations over a one-year period at a USA-based tertiary care academic center to determine the factors predictive of leaving the hospital AMA. The AMA component of these hospitalizations was found to be at 2.7%, which was slightly higher compared to other recently published studies, all of which reported AMA rates of <2% [1,2]. However, it should be noted that at least at our hospital, we have noted a steady increase in the AMA rate following the early COVID-19 period (Geneva et al., unpublished work), and the above-cited studies are based on pre-COVID data.

Based on our at-hospitalization level data and the GEE model, we determined that the most significant predictive factor for an AMA discharge is a personal history of AMA over the preceding 12 months. The adjusted OR was 12, which was more than threefold higher compared to our other top independent variables. This trend had been first reported in a large (nearly 2 million hospitalizations) study in Canada covering the period from 1990 to 2009 [18]. Interestingly, their OR for history of AMA, using a GEE modeling approach similar to ours, was 170. It is important to note that their other significant ORs ranged from 1 to 4, similar to the ones in our study. Therefore, it stands to reason that over the last 20 years, variables other than the history of AMA have increased in importance.

Additional highly significant independent variables from our study, reflected by high ORs in both the person-level and hospitalization-level models, were substance abuse (excluding alcohol), alcohol abuse, and the age groups 35 to 44 years and 25 to 34 years, as noted by many earlier studies [2,3]. However, our study cohort seemed to distinguish itself regarding the fact that patients’ age became a “protective” factor regarding AMA discharge only after age 64. Further, and to our surprise, we found that having a mental health diagnosis did not statistically increase patients’ risk for leaving AMA in the adjusted regression models, even though there was statistical significance in the bivariate analyses. This likely reflects the fact that in the general patient population, other factors play a bigger role and mask the role of mental health. This is certainly not the case when examining purely psychiatric populations (i.e., patients admitted to psychiatry units), where AMA discharge rates had been reported to be in the 3% to 51% range with an average of 17% [3].

Regarding patients’ socioeconomic status, we believe our study is the first to report the association between patients’ SDI (SDI) and the likelihood of AMA discharge. We calculated significant ORs from both the person-level and hospitalization-level models regarding SDI of 60 to 79 and SDI of ≥80. Our findings thus support the application of the SDI in this research field.

The remaining independent variables that demonstrated statistical significance for increased risk for AMA discharge were male sex and the race self-identification (Black/African-American). These had been previously noted to be associated with AMA discharge, but in our study, similar to others’ [2], the OR was not very impressive (<2).

We noted a “protective” effect against AMA discharge among patients whose hospitalization LOS was longer than two days. This seemed reasonable because, as noted above, some of the most important risk factors for AMA discharge are alcohol and substance abuse, and from our clinical experience, AMA is often prompted by improperly controlled withdrawal symptoms, and such symptoms tend to be most intense during the first 48 hours of hospitalization.

Finally, regarding seasonality, we found an evenly distributed AMA discharges across the four seasons, which then obviously resulted in the lack of statistical significance for the bivariate analyses. This likely reflects the fact that the seasonality effect on mood disorders remains debatable despite exhaustive research efforts [19].

There are certain limitations associated with our study. First, we created a single independent variable from all mental health conditions in order to avoid the use of small sample sizes for individual conditions. Therefore, it remains possible that certain individual conditions may have a stronger correlation with AMA than others or the combination of all conditions. However, we must point out that even in Kraut et al.'s study of nearly 2 million hospitalizations [18], statistical significance was barely measurable for individual mental health conditions such as depression. Next, there were certain limitations regarding the data acquisition using the EPIC SlicerDicer data extraction tool. Specifically, we were limited regarding the zip code extraction, and as such, this variable featured the most missing data 14%). Therefore, we ran multiple sensitivity models to ensure that conclusions were not affected. Further, the SDI variable (which was derived using the zip code data) reached statistical significance in our bivariate and multivariate regression models despite the missing data. Finally, and theoretically, there could have been additional independent variables that determine the probability of AMA discharges, but we chose to include the variables most used in previously published research, as well as those that we believe could play a role based on our collective clinical experience.

## Conclusions

AMA discharges remain a challenging problem for hospitals and can have detrimental health consequences for the involved patients. Our study identified the most important risk factors (history of AMA, alcohol and substance abuse, young-to-middle-aged adults, and high SDI). This offers the opportunity for early identification of high-risk patients and for the implementation of prevention strategies, such as the timely addressing of withdrawal symptoms in patients with alcohol abuse and substance dependence. Improvement of patient-centered communication, integration of addiction services and case management teams, as well as clinical decision support alerts from electronic health records, could represent key strategies for the reduction in AMA discharges, thus improving healthcare for vulnerable populations.
